# Prevalence of incidental breast cancer and precursor lesions in autopsy studies: a systematic review and meta-analysis

**DOI:** 10.1186/s12885-017-3808-1

**Published:** 2017-12-02

**Authors:** Elizabeth T. Thomas, Chris Del Mar, Paul Glasziou, Gordon Wright, Alexandra Barratt, Katy J. L. Bell

**Affiliations:** 1 Faculty of Health Sciences and Medicine, Bond University, Robina, QLD 4229 Australia; 2 Centre for Research in Evidence-based Practice, Faculty of Health Sciences and Medicine, Bond University, Robina, QLD 4229 Australia; 3 Sydney School of Public Health, Sydney Medical School, Edward Ford Building (A27), University of Sydney, Fisher Road, Sydney, NSW 2006 Australia

**Keywords:** Breast neoplasms, Mass screening, Early detection of cancer, Autopsy

## Abstract

**Background:**

Autopsy studies demonstrate the prevalence pool of incidental breast cancer in the population, but estimates are uncertain due to small numbers in any primary study. We aimed to conduct a systematic review of autopsy studies to estimate the prevalence of incidental breast cancer and precursors.

**Methods:**

Relevant articles were identified through searching PubMed and Embase from inception up to April 2016, and backward and forward citations. We included autopsy studies of women with no history of breast pathology, which included systematic histological examination of at least one breast, and which allowed calculation of the prevalence of incidental breast cancer or precursor lesions. Data were pooled using logistic regression models with random intercepts (non-linear mixed models).

**Results:**

We included 13 studies from 1948 to 2010, contributing 2363 autopsies with 99 cases of incidental cancer or precursor lesions. More thorough histological examination (≥20 histological sections) was a strong predictor of incidental in-situ *cancer* and *atypical hyperplasia* (OR = 126·8 and 21·3 respectively, *p* < 0·001), but not *invasive cancer* (OR = 1·1, *p* = 0·75). The estimated mean prevalence of incidental cancer or precursor lesion was 19·5% (0·85% *invasive cancer* + 8·9% in-situ *cancer* + 9·8% *atypical hyperplasia*).

**Conclusion:**

Our systematic review in ten countries over six decades found that incidental detection of cancer in situ and breast cancer precursors is common in women not known to have breast disease during life. The large prevalence pool of undetected cancer in-situ and atypical hyperplasia in these autopsy studies suggests screening programs should be cautious about introducing more sensitive tests that may increase detection of these lesions.

## Background

Breast cancer is common [1] and its incidence has been rising [2], largely from increased early detection of cancer through breast screening [3]. For a woman deciding whether or not to undergo mammography screening, the potential benefits of screening include averting the development of advanced breast cancer and possible premature death from breast cancer. However, these benefits must be considered alongside potential harms, one being the overdiagnosis and overtreatment of screen-detected cancers that would otherwise never become apparent during the woman’s lifetime. Cancer overdiagnosis, the diagnosis of cancers which never declare themselves during the patient’s lifetime, may result from the detection of cancers which are very slowly progressive, non-progressive or even regressive, and may include overdiagnosis of both invasive cancer and in-situ cancer [4–13]. Pre-cancer overdiagnosis may also occur where there is detection of lesions such as atypical hyperplasia which either do not advance or do so only very slowly.

Autopsy studies may be used to estimate the size of the prevalence pool of incidental cancer (and pre-cancerous lesions) among people not known to have specific cancers during life. The prevalence pool of incidental prostate cancer, for example, has been estimated as 5% at age < 30 years rising to 59% by age > 79 years [14]; and incidental thyroid cancer as 5·7% overall, and 11·2% when the tissue is examined more intensively [15]. As such, autopsy studies can provide an indication of the potential for overdiagnosis of specific cancers if efforts to detect preclinical cancers and pre-cancers are made. The prevalence pool of incidental breast cancer was investigated in a systematic review of autopsy studies in 1997, which reported a median rate of 1·3% of undiagnosed invasive breast cancer and 8·9% of undiagnosed ductal carcinoma in-situ (DCIS) [16]. Potential breast cancer overdiagnosis remains just as relevant now as then, if not more so. Changes to screening programmes to include the screening of older women (>70 years of age), have resulted in increased numbers of women undergoing screening [17–21], and theoretically could increase overdiagnosis disproportionately [7, 22]. Moreover, increasingly sensitive diagnostic screening technologies have meant increased detection of a number of precursor lesions: *atypical ductal* and *atypical lobular hyperplasia* (ADH and ALH respectively) in addition to *ductal*, and *lobular carcinoma* in-situ (DCIS and LCIS respectively).

In this study we aimed to update the estimated size of the prevalence pool (reservoir) of incidental breast cancer and precursor lesions at autopsy, and identify factors that were associated with increased prevalence of these lesions.

## Methods

### Protocol and registration

The review protocol was not registered.

### Selection

We included autopsy studies of adult women (>age 18 years) who had no history of pre-existing breast disease and which included a systematic histological examination of at least one breast. We excluded studies that did not report the women’s age, or methodically examine the breast microscopically. The principal outcomes were rates of incidental breast cancer (*invasive and* in situ *cancer*), and precursor lesions (*atypical hyperplasia*) diagnosed on histopathology.

### Searching

We searched Medline and Embase using the terms listed below, with no restrictions on year published, type of publication, or language (search terms created by a librarian). To identify further papers for inclusion in the review we ran forward citation searches and checked the references of all papers identified by the search for inclusion in the review. Finally, we repeated our original search to identify any additional papers published during the period of data collection.

#### Search strategy (Medline)

1. exp. Breast Neoplasms/
2. exp. Breast/pa [Pathology]
3. Breast Diseases/pa [Pathology]
4. ((breast or mammary) adj3 (neoplasm* or neoplasia* or tumour* or tumor* or cancer* or carcinoma* or adenocarcinoma* or malignan* or pre-malignan* or premalignan*)).tw.
5. or/1–4
6. Autopsy/
7. (autopsy* or autopsies or postmortem* or post-mortem* or post mortem*).tw.
8. 6 or 7
9. 5 and 8

### Validity assessment

We planned a priori sub-group analyses for the following risk of bias study characteristics: consecutive versus non-consecutive case selection, population based versus hospital based studies, and the possibility that breast cancer discovered at autopsy may have caused death. We also planned subgroup analysis of the following pathology validity characteristics: intensity of pathological examination (average number of sections submitted for histopathology per case); whether the histopathology reporting method used international standards (such as WHO [23]); and peer review of the histopathology diagnosis.

### Study selection and data abstraction

Two authors (KB and ET) independently checked the titles and abstracts of all articles retrieved from Medline and Embase searches, and the full text was obtained if either author judged the article potentially relevant. The same two authors then independently checked all the full text articles for eligibility. Foreign language papers were translated into English using *Google Translate*. Disagreements were resolved through discussion with two further authors (CDM and PG).

Two authors independently extracted data for English papers (KB and ET) and non-English papers (CDM and KB) using standardized forms. We counted only one cancer or precursor lesion diagnosis per woman – in the case where more than one diagnosis was reported we chose the one of the highest grade/stage (i.e. invasive carcinoma > in situ carcinoma > atypical hyperplasia). We also only counted lesions which were not diagnosed during life – where this was uncertain, we were conservative and did not count the lesion. Disagreements were decided through discussion. We extracted data at the study level on: cancer and precursor prevalence, age, year that autopsies were performed, and the validity measures as described above. Where available, we also extracted data at the within-study level for women <70 years and ≥70 years on: cancer and precursor prevalence.

### Quantitative data synthesis

Our main summary measures were the prevalence of previously undiagnosed breast cancer or precursors: *invasive breast cancer*, in-situ *breast cancer* (DCIS and LCIS), and *atypical hyperplasia* (ADH and ALH). We pooled data from all studies using logistic regression models with random intercepts to represent the distribution of underlying cancer/precursor prevalences between different populations. This type of model also allowed for the nested structure of the data for the within-study analysis of age-specific prevalence estimates. We used the model to examine the impact of the validity characteristics on the prevalence estimates. SAS 9.4 was used for all analyses. The NLMIXED procedure was used to build the models, as has been recommended [24].

## Results

**Fig. 1:**
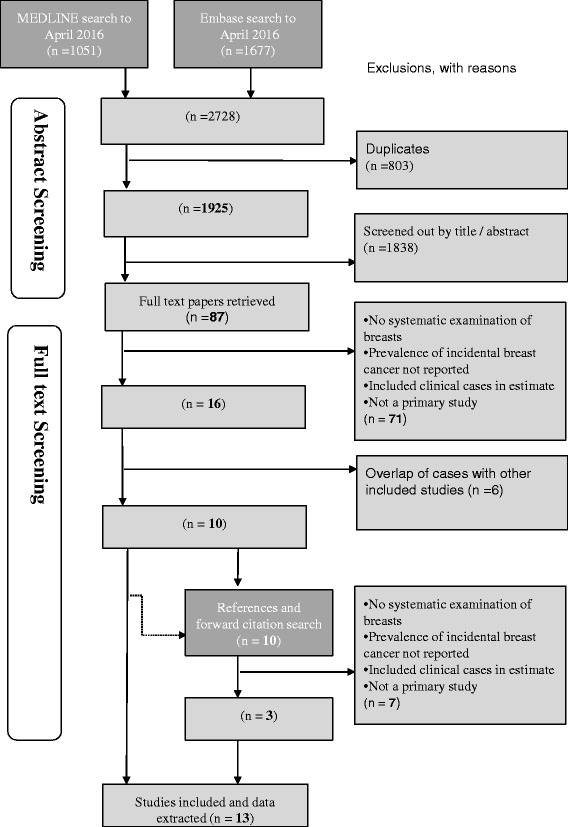
Search and selection of primary studies for the meta-analysis

We identified 1925 abstracts from Medline and Embase (search date 8th April 2016): 87 papers were retrieved for full text review; 71 of which did not meet our selection criteria (Fig. 1). Several of the remaining 16 studies used overlapping data: for each set of potentially overlapping reports, we chose the one that reported on the largest number of women, in the most detail (usually the most recent report), which resulted in 10 studies included from the original search. A further 10 potential papers were identified from the references and forward citation searches of the 10 included papers; of these 3 studies were included, with one [25] contributing two datasets (studies performed in two different countries). A total of 13 studies [25–37], contributing 14 datasets were included in the study level analysis, Table 1. There were 2363 women and 99 cases. There were separate data on cancer and precursor prevalence for women <70 years and ≥70 years in 6 studies [26, 27, 30, 32, 33, 35] for the within-study analysis.

**Table 1:** Estimated prevalence of incidental breast cancer and precursor lesions by year of autopsies done and thoroughness of examination in the 13 included studies

Author	Median year autopsies done	Country/Ethnicity	Number of women	Age (range, years)	Study Population		Peer Review	Used international pathology reporting method	Detected breast cancer possibly caused woman’s death?	Mean number of sections submitted for pathology	Invasive Cancer^a^	In-situ cancer (DCIS^b^/LCIS)^c^	Atypical Hyperplasia (ADH^d^/ALH)^e^	All categories of Cancer or Precursor lesion^a^
					Forensic vs Hospital	Consecutive								
Kiaer	1948	Denmark	351	51	Forensic	No	No	No	No	Not available	0·3%	0·0%	0·0%	0·3%
				(16–89)										
Ryan	1962	Canada	100	56	Hospital	No	Yes	No	Not available	7	0·0%	0·0%	3·0%	3·0%
Sarnelli	1962	Italy	100	62	Hospital	No	No	Yes	No	Not available	0·0%	0·0%	0·0%	0·0%
				(46–90)										
Kramer	1973	USA	70	79	Hospital	No	No	No	Not available	100	1·4%	4·3%	10·0%	15·7%
				(70 –?)										
Nielsen 1984	1977	Denmark	75	67	Hospital	Yes	No	Yes: WHO (1981), Azzopardi	No	189	1·3%	18·7%	4·0%	26·6%
				(22–89)										
Bartow	1981	USA	519	43	Forensic	Yes	No	No	No	18	1·0%	0·4%	1·0%	2·3%
				(15–88)										
Nielsen 1987	1984	Denmark	109	39	Forensic	Yes	No	Yes: WHO (1981), Azzopardi	No	549	0·9%	18·3%	7·3%	26·6%
				(20–54)										
Alpers	1985	USA	101	51	Hospital	No	No	No	No	253	0·0%	8·9%	10·9%	19·8%
				(15–99)										
Pisano	1985	Chile	152	44	Forensic	No	Yes	Yes	No	3	0·7%	0·0%	0·0%	0·7%
Bhathal	1985	Australia	207	60	Forensic	Yes	No	Yes: Black & de Chabon	No	23	1·4%	13·0%	12·6%	27·1%
				(15–97)										
Giarelli	1985	Italy	463	77	Hospital	Yes	No	No	No	Not available	1·5%	0·0%	0·0%	1·5%
				(35 –?)										
Inai	1985	Japan	62	55	Hospital	No	No	Yes: Azzopardi	No	36	1·6%	0·0%	14·5%	16·1%
				(11–85)										
Starlsberg (Ghana)^f^	2010			39	Hospital	No	Yes	No	Yes	8	7·1%	0·0%	0·0%	7·1%
		Ghana	28	(15–60)										
Starlsberg (Norway)^f^	2010			42	Hospital	No	Yes	No	Yes	8	3·8%	0·0%	3·8%	7·7%
		Norway	26	(15–60)										

^a^Prevalences may differ to those reported in primary studies due to exclusion of cases which may have been diagnosed during life

^b^ *DCIS* Ductal carcinoma in-situ

^c^ *LCIS* Lobular Carcinoma in-situ

^d^ *ADH* Atypical Ductal Hyperplasia

^e^ *ALH* Atypical Lobular Hyperplasia

^f^In Stalsberg study, two women with invasive breast cancer first diagnosed at autopsy died of cardiac tamponade/massive pleural effusion and metastatic signet cell carcinoma (the women were from Ghana and Norway respectively)

The median prevalence of: *invasive cancer*; in-situ *cancer* (DCIS and LCIS); *atypical hyperplasia* (ADH and ALH); and any of these lesions; were 1·1% (range 0–7·1%; mean = 1·5%), 0·0% (range 0–18·7%; mean = 4·5%), 3·4% (range 0–14·5%; mean = 4·8%) and 7·4% (0–27%; mean = 10·9%) respectively, Table 1. The overall prevalence of incidental breast cancer or precursor lesion for the studies by the median year the autopsies were done (or year-of-publication if this information was unavailable) showed no visible trend in estimated prevalence over time, from the earliest study in 1947, to the most recent study in 2010, Fig. 2. We formally tested for temporal trend in the models below.

**Fig. 2:**
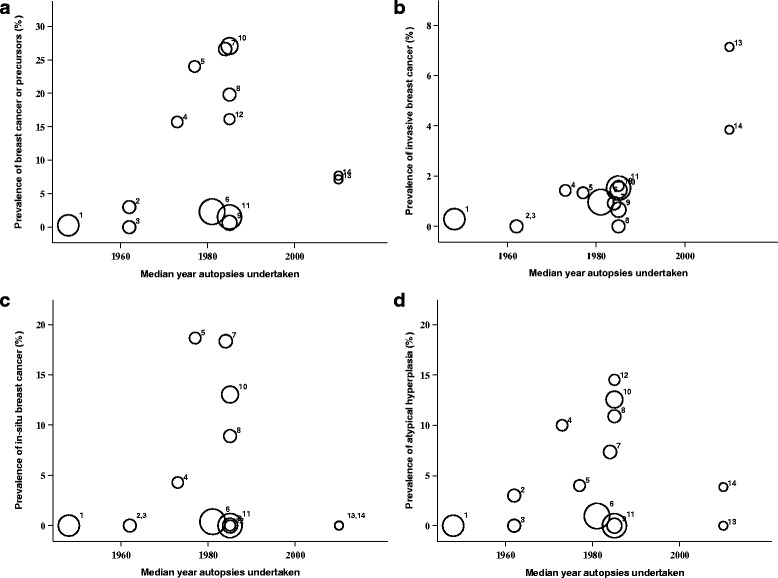
Prevalence of incidental breast cancer or neoplastic precursor lesions in studies by median year of autopsies. 2**a**: Cancer or precursor lesion 2**b**: Invasive cancer 2**c**: In-situ cancer 2**d**: Atypical Hyperplasia. Data points are proportional to total number of women in each study and are numbered according to year of study as follows: 1: Kiaer [26], 2: Ryan [27]; 3: Sarnelli [28]; 4: Kramer [29]; 5: Nielsen 1984 [30]; 6: Bartow [31]; 7: Nielsen 1987 [32]; 8: Alpers [33]; 9: Pisano [34]; 10: Bhathal [35]; 11: Giarelli [36]; 12: Inai [37]; 13: Stalsberg (Ghana) [25]; 14: Stalsberg (Norway) [25]

**Fig. 3:**
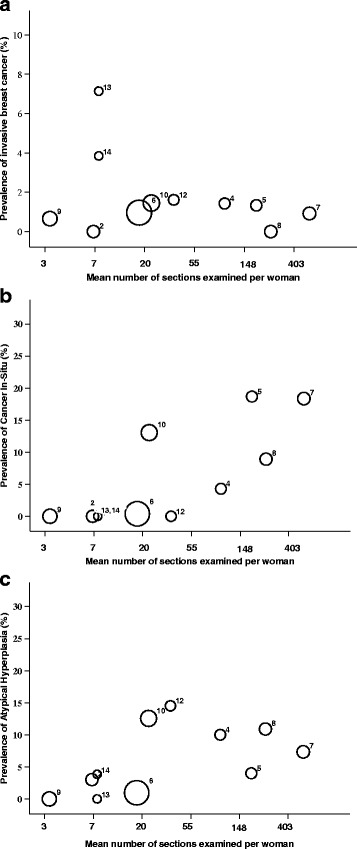
Prevalence of incidental breast cancer or neoplastic precursor lesion in studies by thoroughness of pathology examination. 3**a**: Invasive cancer 3**b**: In-situ cancer 3**c**: Atypical Hyperplasia. Data points are proportional to total number of women in each study and are numbered according to year of study as follows: 1: Kiaer [26], 2: Ryan [27]; 3: Sarnelli [28]; 4: Kramer [29]; 5: Nielsen 1984 [30]; 6: Bartow [31]; 7: Nielsen 1987 [32]; 8: Alpers [33]; 9: Pisano [34]; 10: Bhathal [35]; 11: Giarelli [36]; 12: Inai [37]; 13: Stalsberg (Ghana) [25]; 14: Stalsberg (Norway) [25]

Predictors for incidental prevalence of each category (*invasive cancer*, in-situ *cancer*, *atypical hyperplasia*) and all categories combined, are presented in Table 2. Their prevalence differed between studies (test for random intercepts *p* < 0·001 in each model). More thorough pathology examination yielded greater prevalences of in-situ *cancer*, *atypical hyperplasia* and all categories combined (OR = 126·8, 21·3 and 29·3, if ≥20 sections taken on average compared to <20 sections taken on average respectively), but not *invasive cancer* (OR = 1·1), Fig. 3. The prevalence of each category, and of all categories combined was not statistically higher for studies with an older average age of participants. There was weak evidence for higher prevalence of in-situ *cancer* in studies that used consecutive cases (*p* = 0.06), and of all categories combined for more recent studies (*p* = 0.09), but this disappeared after adjusting for thoroughness of pathology examination (*p* = 0.11 and *p* = 0.37 respectively). There was a higher prevalence of *invasive cancer*, (but not in-situ *cancer* or *atypical hyperplasia*), for the study (contributing two datasets) [25] reporting breast cancer discovered at autopsy which may have contributed to the women’s deaths. None of the other available validity characteristics were statistically significant.

**Table 2:** Estimates of association (from logistic regression model) of potential predictors for prevalence of breast cancer or precursor lesion

Possible Predictor	Invasive Cancer (IC)		In-situ cancer (DCIS^a^ + LCIS^b^)		Atypical Hyperplasia (ADH^c^ + ALH^d^)		All categories (IC + DCIS + LCIS + ADH + ALH)	
	*Odds Ratio*	*P Value*	*Odds Ratio*	*P Value*	*Odds Ratio*	*P Value*	*Odds Ratio*	*P Value*
Mean number of histologic sections examined per woman		0·75		<0·001*		<0·001*		0·002*
per unit on logscale	0.9 (0.7–1.3)		3·2 (1·6–6·4)*		1·6 (1·1–2·4)*		1·79 (1·3–2·4)*	
> 20 sections taken on average	1·1 (0·5–2·7)		127.0 (20·2–793)*		21·3 (9·7–46·7)*		22·4 (17·2–49·8)*	
Mean age		0·48		0·75		1·0		1·00
per decade increase	1.1 (0.8–1.5)		1·3 (0·34–5·30)		1·1 (0·4–2·7)		1·07 (0·53–2·1)	
Mean age > 50	0·8 (0·3–1.7)		1·9 (0·03–105.1)		2·2 (0·2–25·7)		1·24 (0·08–7·9)	
Mean age > 70	1.7 (0.7–4.1)		0·6 (0·003–4·6)		0·5 (0·02–13·1)		0·91 (0·08–2·4)	
Forensic vs hospital based	0.6 (0.3–1.4)	0.27	2·8 (0·05–168.0)	0·53	0·5 (0·05–5·7)	0·65	0·60 (0·10–3·7)	0·58
Consecutive cases	1.8 (0.7–4.3)	0.19	37·7 (0·5–2777.0)	0·06	1·5 (0·1–16·3)	1·0	2·3 (0·48–11·2)	0·32
Adjusted for number of sections examined			4·5 (0·77–26·3)	0·11				
Breast cancer may have caused death	6·6 (1·9–23·3)*	0·02*	0·0 (0·0-∞)	0·21	0·5 (0·0–23·1)	0·75	1·2 (0·1–17·1)	1·00
Peer Review	1·4 (0·5–4.0)	0.58	0·33 (0·04–2·5)	0·29	0·3 (0·02–4·3)	0·44	0·39 (0·06–2·8)	0·37
Internationally recognized reporting method?	1.0 (0.4–2.3)	1.0	8·6 (0·26–288)	0·37	8·6 (0·26–288)	0·37	2·3 (0·42–12·4)	0·37
Time trend (decade of autopsies)	1·0 (0·5–2·0)	1·0	1·0 (0·2–4·4)	0·65	1·4 (0·7–3·0)	0·40	1·6 (0·93–2·7)	0·09
Adjusted for number of sections examined							1·2 (0·81–2·5)	0·37

*P value <0·05

^a^ *DCIS* Ductal carcinoma in-situ

^b^ *LCIS* Lobular Carcinoma in-situ

^c^ *ADH* Atypical Ductal Hyperplasia

^d^ *ALH* Atypical Lobular Hyperplasia

The mean prevalence of invasive cancer was 1·5%. After excluding the study in which the breast cancer discovered at autopsy may have contributed to death [25], it was 0·85%. The mean prevalence of in-situ *cancer* and *atypical hyperplasia*, with modelled adjustment upwards for less thorough studies, was 8·9% and 9·8% respectively. The overall mean cancer and precursor prevalence, with adjustment of in situ and precursor estimates upwards for less thorough studies, (no adjustment for invasive cancer), was 19·5% (0·85% + 8·9% + 9·8%).

When we repeated the analysis, limiting it to six studies with separate data on women <70 years and ≥70 years, there were insufficient data to perform a direct within-study comparison while adjusting for thoroughness of pathology examination.

## Discussion

Our systematic review of 13 studies (14 datasets) in ten countries over six decades of 2363 autopsies and 99 cases of incidental cancer or neoplastic precursor lesions, found that incidental breast cancer and its precursors are common in women not known to have breast disease during life. The estimates from these autopsy studies represent the best available evidence to answer the important question on the size of the prevalence pool of incidental breast cancer and precursor lesions.

The majority of incidental lesions appear to be precursors for invasive cancer (*cancer* in-situ and *atypical hyperplasia*). However the smaller prevalence pool of incidental invasive cancers appeared easier to find, with even the least thorough studies reporting cases. Unlike *invasive cancer* which was unrelated to the number of sections submitted for pathology examination, the estimated prevalence of *cancer* in-situ and *atypical hyperplasia* was strongly related to the thoroughness of microscopic examination. The odds of finding in-situ *cancer* and *atypical hyperplasia* were 127 times and 21 times higher respectively, in the studies where at least 20 sections were examined compared to those where less than 20 sections were examined. We could find no other clear predictors, including age, although this may be attributed to a paucity of data on women ≥70 years.

Our study builds on the evidence from a previous systematic review of incidental cancer discovered at autopsy [16]. We included six of the seven studies in that review (we excluded one study [38] as those data were included in a later report [33]). Our sensitive search strategy uncovered a further six reports which were published at the time of the previous review, but not discovered by them. We also found one more recent study [25] which contributed 2 datasets. Pooling these data enabled us to report on the substantial prevalence pool of invasive cancer, situ-cancer and precursor lesions (ADH and ALH), and that the more thorough the microscopic examination, the more these lesions are discovered.

Autopsy rates are now much lower than previous decades; in addition, the widespread adoption of screening in many countries means that contemporary studies risk under-estimating the prevalence of incidental disease as much of this may have already been detected (and treated) during life. Although the primary studies were conducted over a long time period (from 1948 to 2010), all but one were conducted in largely unscreened populations. The most thorough studies were conducted in the 1970s and 1980s, and included both hospital and forensic studies.

Limitations to this review include variation in the prevalence of incidental breast cancer and precursors across the studies, which could in part be due to underlying differences within the populations studied. Pathologists may have differing thresholds for classifying lesions [39] and differing levels of scrutiny with which they analyse lesions - which, as already discussed, was the strongest predictor of incidental breast cancer in-situ and atypical hyperplasia. Our review was also limited by the absence of data on the age-specific prevalence in most of the studies. We compared older and younger women's prevalences of incidental cancer and precursors but had insufficient data to make any conclusions on this. Insufficient information also prevented us from being able to compare cancer prevalence across race groups. In particular, there was a paucity of data related to women of African descent (only one study).

The size of the prevalence pool of incidental invasive breast cancer in unscreened populations may be used to provide an approximate lower bound for the extent of overdiagnosis associated with mammography screening: true overdiagnosis rates are likely to be at least this large. Our estimate of the prevalence of incidental invasive cancer, at 0·85%, is much less than the current life-time prevalence of invasive cancer for women in the USA of 12·4% [40], or the life-time prevalence of screen-detected invasive cancer of ~7·4% (assuming that about 60% of invasive cancers diagnosed during life are detected by mammography screening [41, 42]). If we assume that all of the prevalence pool of incidental cancer would be detected through screening (which is reasonable given the apparent ease with which incidental invasive cancer was detected in the autopsy studies), then the implications may be that at least ~11% (~0·85/7·4%) of screen-detected invasive cancers, or at least ~7% (~0·85/12·4) of all invasive cancers, are overdiagnosed. Invasive cancers that would have regressed if not detected by screening [43, 44] however, will cause the lifetime prevalence of overdiagnosed cancers to be greater than the incidental cancer prevalence discovered at autopsy in unscreened populations.

The excess lifetime prevalence of breast cancer in a regularly screened population may be used to provide an approximate upper bound for the extent of overdiagnosis associated with mammography screening: true overdiagnosis rates are likely to be no larger than this. The lifetime prevalence of invasive breast cancer in the USA in 1975–1977 (prior to the introduction of screening) was 9.4% (1 in 10.6 women). Since 1987 after roll-out of nation-wide mammography screening the life time prevalence has been stable at around 12.5% (1 in 8 women). Some of the increased risk in more recent times is because women are now less likely to die of other causes and because of changing risk factor levels, but the main explanation appears to be increased detection through mammography screening [45]. The expected decline in lifetime prevalence as screening rates stabilized has not eventuated [46] and a large proportion of the excess 3% lifetime prevalence (12.4% - 9.4%), which has now persisted for 30 years, is likely to be due to overdiagnosis. The implications of this are that up to ~40% (~3%/7.4%) of screen-detected invasive cancers, or up to ~24% (~3/12.4%) of all invasive cancers, may be currently overdiagnosed. Others’ estimated overdiagnosis rates fall between our approximate lower and upper bounds [11, 43, 47–50].

For in-situ breast cancer, our estimate for the prevalence pool of incidental lesions is ~9%, much higher than the current life-time prevalence of ~2·0% [40], and lifetime prevalence of screen-detected in situ cancer of 1.6% (approximately 80–85% of in-situ cancers diagnosed during life are detected by screening [51]). This means there is a much higher probability of screen detected in-situ cancers being overdiagnosed (perhaps most are overdiagnosed), again consistent with estimates using other methods [11, 47].

The large pool of undetected *cancer* in-situ and *atypical hyperplasia* in these autopsy studies suggest caution for screening programs. First, as new breast screening technologies become more sensitive (e.g. digital mammography and breast tomosynthesis), it is likely that the proportion of overdiagnosed women will increase. Protocols for more intense biopsy sampling of screen detected abnormalities, or enhanced biopsy methods such as stereotactic vacuum-assisted core biopsy, are also likely to further increase overdiagnosis rates. Accordingly, new technologies and biopsy protocols should evaluate whether any increased sensitivity is for clinically important or overdiagnosed cancers, for example by examining interval cancer rates in randomised comparisons of alternative screening technologies [52]. Second, expansion of mammography screening programs to include those aged ≥70 years may also increase the risk of overdiagnosis and overtreatment [22]. The consequences of overtreating older women may also be more serious than for younger women because of their increased susceptibility to adverse effects of treatment [53].

## Conclusion

This review has confirmed that there is a large prevalence pool of incidental breast cancer and precursor lesions present across all ages. Policy-makers, researchers, clinicians and women invited to undergo screening need to be aware of the extent of overdiagnosis so breast cancer detection can be improved and women do not undergo treatment for an inconsequential finding detected through breast cancer screening.

## Abbreviations

ADH: atypical ductal hyperplasia
ALH: atypical lobular hyperplasia
DCIS: ductal carcinoma in-situ
LCIS: lobular carcinoma in-situ
OR: Odds Ratio
USA: United States of America
WHO: World Health Organisation
